# DNA Interaction, DNA Photocleavage, Photocytotoxicity In Vitro, and Molecular Docking of Naphthyl-Appended Ruthenium Complexes

**DOI:** 10.3390/molecules27123676

**Published:** 2022-06-08

**Authors:** Xia Hu, Qian Luo, Yao Qin, Yao Wu, Xue-Wen Liu

**Affiliations:** Hunan Provincial Key Laboratory of Water Treatment Functional Materials, Hunan Province Engineering Research Center of Electroplating Wastewater Reuse Technology, College of Chemistry and Materials Engineering, Hunan University of Arts and Science, Changde 415000, China; huxia@huas.edu.cn (X.H.); luoqian@st.huas.edu.cn (Q.L.); qy20201007@st.huas.edu.cn (Y.Q.); wy1619@st.huas.edu.cn (Y.W.)

**Keywords:** ruthenium complex, DNA interaction, photocleavage, molecular docking, photocytotoxicity

## Abstract

With the development of metal-based drugs, Ru(II) compounds present potential applications of PDT (photodynamic therapy) and anticancer reagents. We herein synthesized two naphthyl-appended ruthenium complexes by the combination of the ligand with naphthyl and bipyridyl. The DNA affinities, photocleavage abilities, and photocytotoxicity were studied by various spectral methods, viscosity measurement, theoretical computation method, gel electrophoresis, and MTT method. Two complexes exhibited strong interaction with calf thymus DNA by intercalation. Production of singlet oxygen (^1^O_2_) led to obvious DNA photocleavage activities of two complexes under 365 nm light. Furthermore, two complexes displayed obvious photocytotoxicity and low dark cytotoxicity towards Hela, A549, and A375 cells.

## 1. Introduction

The biological properties of ruthenium metal complexes are of immense interest to many biochemists due to their importance in some biochemical events, such as luminescent cellular imaging, luminescent probe, antibacterial activity, antitumor activity, and phototherapeutic ability, etc. [1,2,3,4,5,6,7,8]. Many studies have shown that these potential biological applications may be attributed to the excellent properties of ruthenium complexes, such as binding to DNA or protein, good luminescent behaviors, and singlet oxygen generating abilities [9]. Hence, the design of ruthenium complex containing new ligands with different structures may create desirable biological activities.

Most PDT agents can generate ROS after light activation, leading to DNA damage and killing target cells. Photofrin^®^ is the first PDT clinical drug based on porphyrin compound, which showed excellent photocytotoxicity against solid tumors [10]. Ruthenium-based compounds usually exhibit high singlet oxygen quantum yields [11,12,13,14,15,16,17]. Most of them have been reported as efficient photodynamic therapy (PDT) and photochemotherapy (PCT) agents and display remarkable antitumor activities, which provokes interests of more and more scientists [11,12,13,14,15,16,17].

Naphthyl moiety has shown its importance in developing chemosensors and drugs with chemotherapeutic activities [12,13]. Naphthyl unit is a typical fluorophore, which improves the emission properties of organic molecules and their biological activities, such as DNA topoisomerase inhibition, antitumor agents, and antibacterial activities [10,11,12,13,14,15,16,17,18,19]. Naphthyl moiety is also introduced into the ligand on metal complexes, which exhibits excellent biological properties [14,15,16,17]. Recently, several ruthenium complexes containing naphthyl moiety were synthesized and their biological activities were tested [11,15,16,17,18,19]. For example, Sousa and Carvalho reported that two aryl-substituted ruthenium(II) complexesexhibitedhigh quantum yields for singlet oxygen and strong DNA affinities by using a ligand containing naphthyl group as co-ligand and a typical intercalative ligand (dppz, dipyridophenazine) as main ligand [18]. Howwever, these complexes have not been utilized to investigate the PDT activities. Greer and McFarland synthesized ruthenium complexes with a π-expansive imidazophen ligand by the link between a naphthyl group and 1H-imidazo[4,5-*f*][1,10]phenanthroline [16]. Ru(II) complex displayed high singlet oxygen quantum yield and phototherapeutic index (PI). The introduction of the naphthyl unit was found to enhance ^1^O_2_ quantum yields. They also found that the complexes containing phenyl group exhibited larger PI value (558)compared to the introduction of naphthyl group, though the complex containing naphthyl group displayed higher singlet oxygen quantum yield than the complex containing phenyl group. Thus, singlet oxygen quantum yield is not only factor that affects PDT activities of ruthenium complexes. Combined with previous reports [11,12,13,14,15,16,17], DNA affinity is also an important factor for PDT activity, since DNA is the target for PDT agents. [Ru(bpy)_3_]^2+^ displayed high singlet oxygen quantum yield, but exhibited low PI, since it has weak DNA affinity [20]. Furthermore, [Ru(bpy)_2_dppz]^2+^ (dppz = dipyrido[2,3-a:3′,2′-c]phenazine) binds to DNA strongly, but has low singlet oxygen quantum yields, which leads to low photocytotoxicity [21]. Although Greer and McFarland have not tested DNA affinity of the complex containing naphthyl group and the complex containing phenyl group, an early report has shown that introducing a large naphthyl group results in low DNA affinity due to the possible steric hindrance [22]. This may be the cause for the lower PI value of the complex containing naphthyl group in Greer and McFarland’s case. Therefore, introducing naphthyl units into ruthenium complexes to keep high singlet oxygen yields and strong DNA affinities may endow them with possible enhanced PDT activities.

**Scheme 1:**
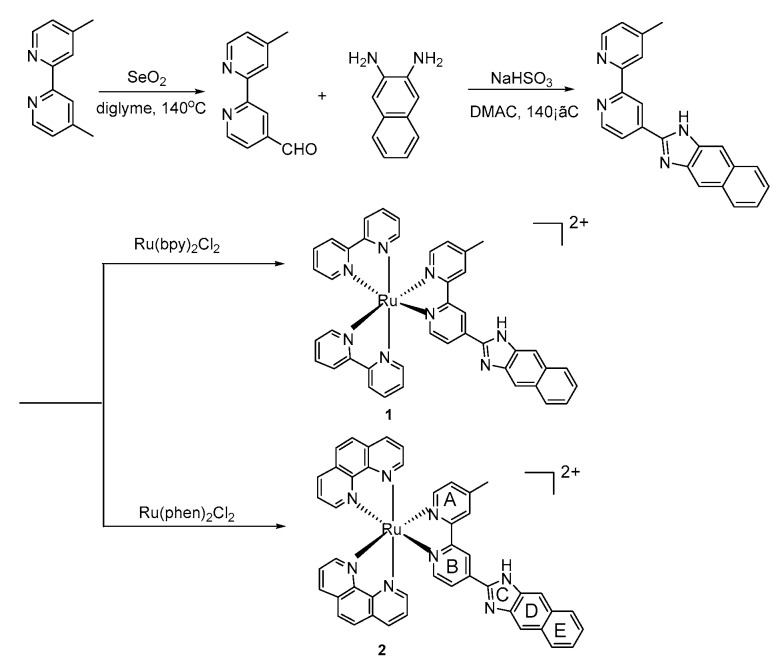
Synthesis routine of ligand mbin and its ruthenium complexes.

In this context, we linked bipyridyl with the rigid naphthyl substituent by an imidazol ring and obtained a new main ligand, mbin (2-(4′-methyl-bipyridine-4-yl)-1H-imidazo[4,5-b]naphthalene), and two ruthenium complexes (Scheme 1, Figures S1 and S2). Synthesized ruthenium complexes were used to study the DNA binding behaviors, DNA photocleavage, and photocytotoxicity in vitro.

## 2. Results and Discussion

### 2.1. Studies on DNA Interaction

In the PDT processes, DNA is one of the potential target molecules in cancer. The affinity strength of the DNA bound by ruthenium complexes can affect DNA cleavage and the PDT efficiency in cancer cells. Commonly used methods to measure DNA affinity between small molecules and double-strand DNA include absorption titration, emission titration, emission EB displacement experiment, viscosity measurement, and so on.

**Figure 1:**
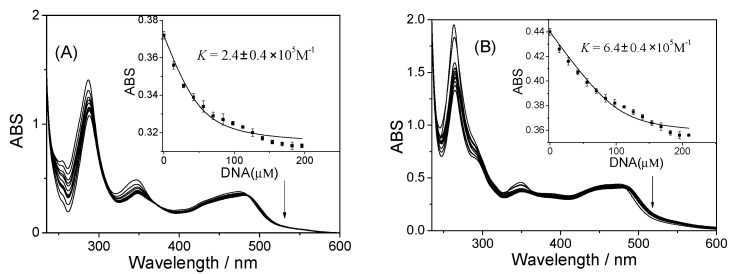
Absorption spectra of **1** (**A**) and **2** (**B**) (20 μM) after titrating different amounts of CT-DNA. Inset: plots of *ε_a_* vs. [DNA].

Herein, UV-vis spectra were measured by titrating CT-DNA (CT = calf thymus) into the solution of ruthenium complexes. The spectra in UV-vis region were shown in Figure 1. From Figure 1, the absorptivity decreased with increasing DNA concentrations for both complexes. The MLCT band localized at 474 or 473 nm for **1** and **2**, respectively. The change in absorption intensity at MLCT band was utilized to calculate DNA binding constant, ***K***, using McGhee–von Hippel Equation (1a,b) [23]. Previous studies reported that the value of ***K*** for typical intercalator, [Ru(bpy)_2_dppz]^2+^, was (***K*** = 4.9 × 10^6^ M^−1^) [24,25]. [Ru(bpy)_3_]^2+^ binds to CT-DNA through electrostatic mode with the value of ***K*** as 0.7 × 10^3^ M^−1^ [26]. The values of ***K*** are 2.4 ± 0.4 × 10^5^ M^−1^ (s = 1.25 ± 0.21) and 6.4 ± 0.3 × 10^5^ M^−1^ (s = 2.63 ± 0.24), for complex **1** and **2**, respectively. Here, for complex **2**, the binding site size is about 2 base pairs. However, the value of s for complex **1** is less than that of complex **2**, which could indicate that the complex molecules stacked each other on the DNA surface, according to previous reports [26]. The results showed that our complexes displayed higher DNA affinities than that of [Ru(bpy)_3_]^2+^, but lower DNA affinities than that of [Ru(bpy)_2_dppz]^2+^.

**Figure 2:**
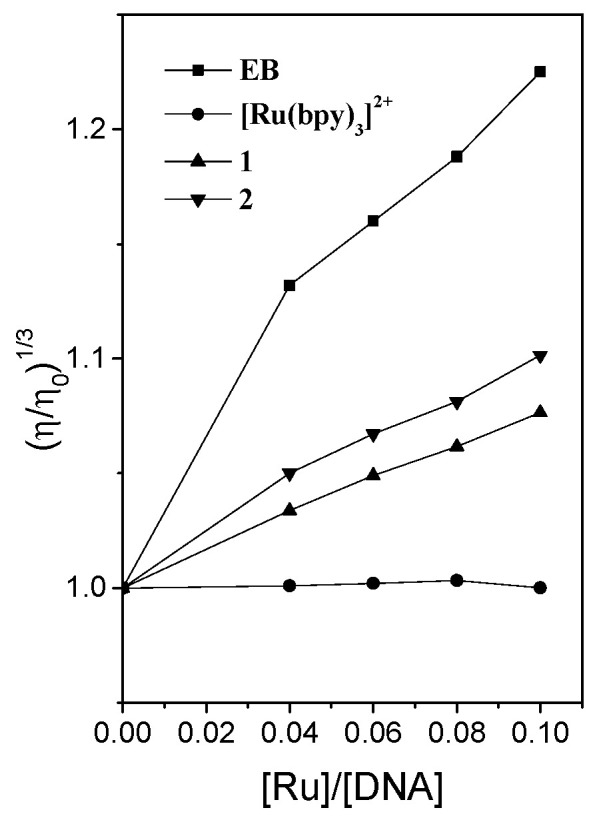
The viscosity of DNA (0.25 mM) after incubation with different concentrations of **1** (▲), **2** (▼), [Ru(bpy)_3_]^2+^ (●), and EB (■).

Viscosity experiments were performed using an Ubbelohde viscometer to demonstrate whether the complexes intercalate into base pairs. When small molecules intercalate into base pairs of DNA, DNA length will increase, which leads to DNA viscosity increase. The electrostatic binding compounds cannot change the length of DNA and display no obvious change in DNA viscosity. Therefore, the change in DNA viscosity can distinguish different binding modes against CT-DNA. Here, EB and [Ru(bpy)_3_]^2+^ were selected to be the controls for intercalator and electrostatic binder [27,28,29], respectively. Obvious increase in DNA viscosity was observed for EB (Figure 2), indicating EB intercalates into base pairs of CT-DNA. Little change in DNA viscosity was consistent with the previous reported result for [Ru(bpy)_3_]^2+^, and electrostatic binding mode was assigned to [Ru(bpy)_3_]^2+^ [27,28,30]. For synthesized Ru((II) compounds, the increase in DNA viscosity was observed as same as that of EB, indicating that two complexes present a similar binding process to EB. The results designated DNA intercalative binding mode for two complexes. Larger increase in DNA viscosity indicated that interaction with **2** is larger than **1**. Ruthenium polypyridyl complexes containing auxiliary ligand with larger hydrophobicity usually displayed higher DNA affinity. Here, complex **2** contains auxiliary ligand (phen) with larger hydrophobicity compared to complex **1** (bpy), which results in larger DNA affinity [30].

**Figure 3:**
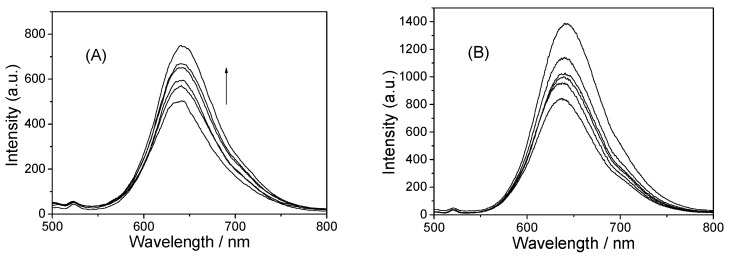
Luminescence spectra of **1** (**A**) and **2** (**B**) (5 μM) after binding to CT-DNA.

Emission titration experiments were also used to prove strong DNA interaction with two complexes by adding DNA into complex solutions. The results were shown in Figure 3. Upon excitation at 450 nm, obvious luminescence was observed for **1** and **2** at 636 and 648 nm, respectively. In the presence of CT-DNA (0–120 μM), the emission spectra showed enhanced luminescence with the *I*/*I*_0_ ratio of 1.46 or 1.75 for **1** and **2**, respectively. This indicated that they bind to DNA strongly. The π-π stacking interactions between ruthenium complexes and base pairs restrict the mobility of ruthenium complexes and lead to luminescence enhancement [31].

**Figure 4:**
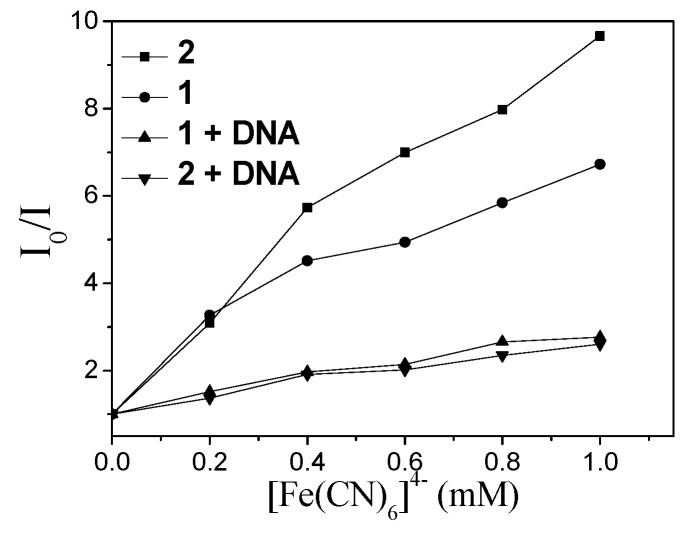
Luminescence quenching curves of **1** (●), **2** (■), **1**-DNA (▼), and **2**-DNA (▲) by [Fe(CN)_6_]^4^^–^. [Ru] = 5 μM, [DNA]/[Ru] = 80.

To further investigate the strong DNA affinity of two complexes, [Fe(CN)_6_]^4−^ was used to quench the luminescence of the complexes with or without DNA. [Fe(CN)_6_]^4−^ is a tetra-charged anion, which may bind to ruthenium complex cations via electrostatic attraction [32]. Thiswill lead to the decrease in the luminescence of the complexes. Protection of ruthenium complex cation by DNA against [Fe(CN)_6_]^4−^ will prevent luminescence quenching, which depends on the degree of protection by DNA [32]. The emission spectra of two complexes without or with DNA were collected in the range of 500 to 800 nm (Figure 4). In the presence of 0–1 mM [Fe(CN)_6_]^4−^, the luminescence intensities of the complex solutions decreased sharply. In the presence of DNA, the degree of luminescence quenching obviously decreased, indicating that DNA efficiently protected ruthenium complexes from [Fe(CN)_6_]^4−^, and strong interactions existed between the two complexes and DNA.

**Figure 5:**
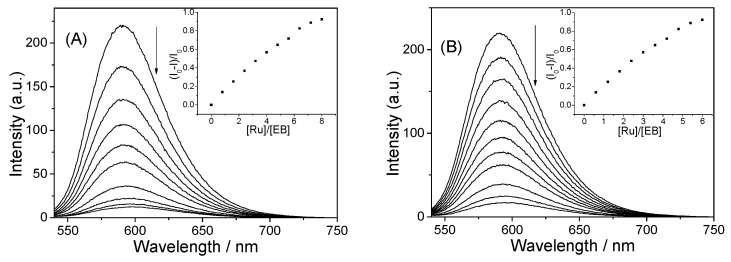
Luminescence spectra of EB-DNA system after adding ruthenium complexes. (**A**) **1** and (**B**) **2**.

Ethidium bromide (EB) competitive displacement assay was also used to confirm the intercalative binding mode. EB is a commonly used DNA fluorescence dye that binds to DNA through intercalation. EB cannot emit in water and DNA-bound EB can emit strong fluorescence at 600 nm. The assay has been implemented by competitive displacing EB from EB-DNA mixed system. The degree of decrease in fluorescence intensities may reflect intercalative binding mode and DNA affinity compared to EB. As shown in Figure 5, EB-DNA mixed solutions emitted strong fluorescence at 597 nm. The addition of the complex solutions into EB-DNA mixed solution, caused the fluorescence of EB-DNA system to be quenched, indicating that our compounds intercalated into base pairs and displaced EB from DNA. Almost 91% and 92% quenching efficiencies for complexes **1** and **2** also showed that two complexes displayed lower DNA affinities than that of EB and can displace EB from DNA. The DNA affinities were also determined using emission data from EB competitive displacement assay. The DNA binding constants (*K*_app_) were calculated by LePecq equation [33]. Using *K*_EB_ (1.4 × 10^6^ M^−1^) as the control [34,35], the binding constants calculated were 4.38 × 10^5^ M^−1^ and 5.42 × 10^5^ M^−1^ for **1** and **2**, respectively. The *K*_app_ values were similar to those obtained from absorption titration results.

**Figure 6:**
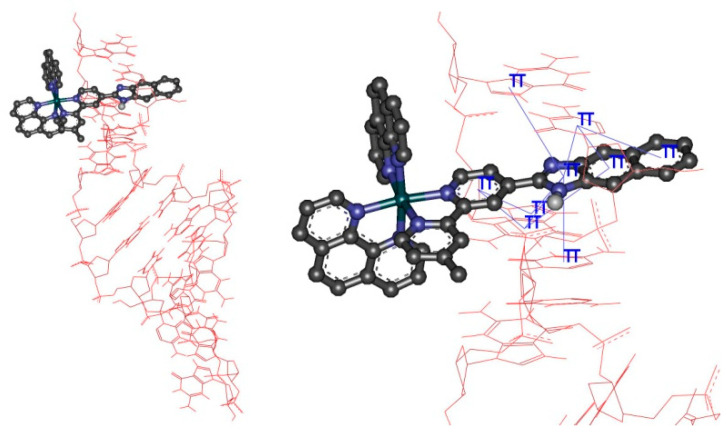
The ds-DNA (PDB: 4E7Y) binding conformation of complex **2** by docking.

Molecular docking was performed to further explore the factors affecting the interaction between two complexes and double-strand DNA (PDB: 4e7y) (Figure 6). The ground state geometries optimized by Gaussian 09 (Figure S3) were docked into DNA. The docking poses were ranked by the calculated binding energies. The best conformation of ligand-DNA model was determined by choosing the docking pose with the minimum binding energy. For complex **2** and **1**, the minimum double-strand DNA binding energy were observed as −5.8 and −1.9 kcal/mol, respectively. The smaller DNA binding energy of complex **2** indicated that complex **2** may bind more strongly to DNA than complex **1**. From the docking results, we also found that two complexes intercalated into base pairs by using the same main ligand (mbin). In addition, the π-π stack interactions were found between B, C, D, and E ring and base pairs of DNA for two complexes (Figure 6 and Figure S4), which was the main contribution in the DNA binding process. Although obvious π-π stack interactions were observed from the docking results, the difference in the π-π stack interactions between two complexes and DNA cannot be obtained by Autodock software. Further studies are underway to explore the difference in the interactions with DNA.

### 2.2. DNA Photocleavage Activities

**Figure 7:**
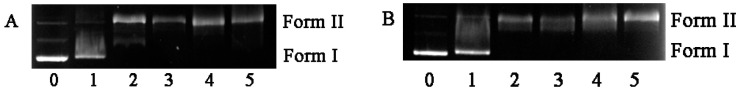
DNA photocleavage of pBR322 DNA dosed with complexes **1**, **2**, and irradiation (365 nm) for 1.5 h. Lane 0, DNA; Lane 1, DNA + Ru, no hv; Lane 25: **1** (**A**) at 20, 40, 60, and 80 μM; **2** (**B**) at 10, 20, 30, and 40 μM.

As described above, PDT agents can produce ROS after photoactivation and damage DNA. Here, we used a plasmid DNA as the probe to test the photobiological activity of two complexes under 365 nm light. Generally, single strand or double strand breaks can convert the plasmid DNA from supercoil DNA to other forms of DNA [36,37]. The agarose gel electrophoresis results were showed in Figure 7. For DNA without complexes, irradiation did not lead to DNA cleavage. In the presence of the complexes (10–80 μM), two complexes cleaved pBR 322 DNA, and 20 μM complex **2** almost converted supercoil DNA into nicked form (Form II). Compared to complex **1**, complex **2** displayed higher photocleavage efficiency against plasmid DNA due to its stronger DNA binding affinity.

**Figure 8:**
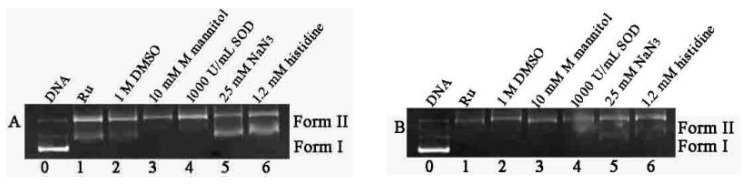
The mechanism experiments of DNA photocleavage. (**A**) complex **1** (20 μM), and (**B**) **2** (10 μM) incubating with different scavengers under 60 min irradiation (365 nm).

The possible reactive oxygen species (ROS) were determined by the mechanism experiments using different scavengers, such as DMSO (DMSO = dimethyl sulfoxide), histidine, sodium azide, mannitol, and SOD (SOD = superoxide dismutase) [37,38,39]. Incubation of NaN_3_ or histidine with Ru(II) complex and DNA mixed solution under irradiation resulted in the decrease in the amount of Form II, and the DNA photocleavage was inhibited (Figure 8). However, other scavengers did not inhibit the DNA photocleavage. Combined with previous results, the DNA photocleavage abilities may be attributed to the singlet oxygen (^1^O_2_) quantum yield for complexes **1** and **2**.

**Figure 9:**
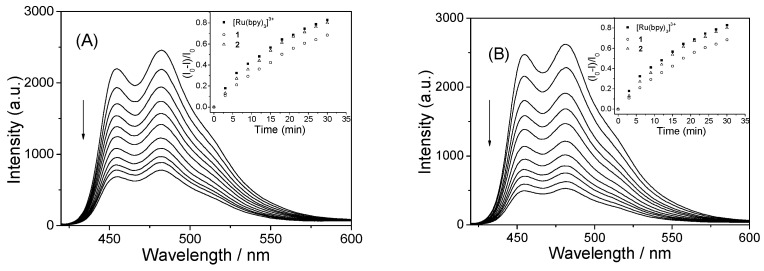
The change on the fluorescence of Ru(II)-DPBF system (**1** (**A**) and **2** (**B**)).

Therefore, the singlet oxygen quantum yield experiments were also performed by testing the fluorescence intensities of DPBF-Ru (DPBF = 1,3-Diphenylisobenzofuran) methanol solutions under 450 nm light. DPBF displays strong fluorescence in methanol. Incubation of DPBF with the sensitizers which produce ^1^O_2_ could result in fluorescence quenching of DPBF. The results were given in Figure 9. The obvious decrease in fluorescence intensities of DPBF-Ru system indicated two complexes produced singlet oxygen under irradiation. The plot of *I*_0_/*I* against irradiation time also showed that a higher singlet oxygen yield was observed for **2** compared to **1**, which is beneficial proof for higher photocleavage ability of complex **2**. Using [Ru(bpy)_3_]^2+^ as the control (=0.81) [40], the ^1^O_2_ generation quantum yields (*Φ*_Δ_) werecalculated according to literature method. The values of *Φ*_Δ_ for complexes **1** and **2** were 0.66 and 0.78, respectively. Two complexes were demonstrated to yield singlet oxygen, which resulted in DNA photocleavage.

### 2.3. Photocytotoxic Activity In Vitro

As mentioned above, the excellent photochemistry properties, strong DNA affinities and high singlet oxygen quantum yields of Ru(II) complexes have attracted many researchers to treat several types of tumors through the photodynamic method. Here, we checked the photocytotoxicities of two complexes and cisplatin by MTT method. HeLa, A549, and A375 cells were used in the experiments. For comparison, dark cytotoxicities in vitro were also measured. Table 1 showed the IC_50_ values of complex **1**, **2**, and cisplatin under irradiation or in the dark. The well-known metal-based anti-tumor drug, cisplatin, was used as the control. It usually displays dark cytotoxicities for many tumors, according to previous reports [4,41]. Here, we also found that cisplatin had no obvious enhanced cytotoxicities under 450 nm irradiation compared to its dark cytotoxicities, which indicated this drug did not produce enough ROS. For the two complexes, obvious photocytotoxicities were observed after 10 min irradiation at 450 nm and 36 h incubation. Interestingly, the two complexes displayed low dark cytotoxicities. Generally, an excellent PDT reagent usually possesses a large PI (photocytotoxicity index) value, meaning high photocytotoxicity and low dark cytotoxicity. The results indicated that two complexes showed potential application for PDT drugs.

**Figure 10:**
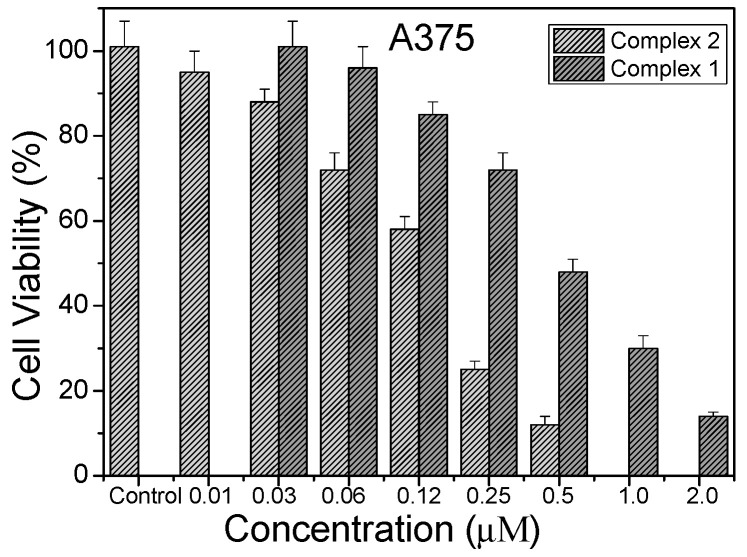
Photocytotoxic effects of **1** and **2** on A375 cells after 12 h incubation and 10 min irradiation.

The PI values for the two complexes are listed in Table 1. The largest PI value was found for complex **2** after treatment with A375 cell. Figure 10 showed the cell viabilities of A375 cells after incubation with complex **1** and **2**. Furthermore, compared with **1**, complex **2** displayed larger PI values against all cancer cells. Generally, two factors contribute to the PDT efficiencies for Ru(II)-based compounds, ROS quantum yields and DNA affinity. Strong DNA affinity usually leads to high DNA cleavage efficacy. Here, **2** displayed stronger DNA affinity and higher ^1^O_2_ quantum yields compared to **1**. The previous reports also demonstrated that the rigidity and planarity of ancillary ligand could favor enhancement of DNA affinity and ROS yields of ruthenium complexes. Complex **2** possesses larger co-ligand (phen) compared to bpy of complex **1**. In previous reported ruthenium complexes, photocytotoxicity of [Ru(bpy)_3_]^2+^ was also used to compare with that of our complexes [20]. This complex displayed high ^1^O_2_ quantum yield, but weak DNA affinity, which has been regarded as a classical DNA electrostatic binding reagent. Therefore, the low photocytotoxicity can be believed to come mainly from its low DNA affinity. After improving the planar area of main ligand by introducing pyrenyl group, [Ru(bpy)_2_(ippy)]^2+^ displayed high ^1^O_2_ quantum yields and strong DNA binding affinity, which resulted in large PI value (413) towards HL-60 cells [20]. Similarly, [Ru(bpy)_2_dppn]^2+^ also exhibited large PI value against HeLa cells [42].

## 3. Materials and Methods

### 3.1. Instrumentation

^1^H NMR and ^13^C NMR signals were determined on a Brucker Advance 400 MHz spectrometer. The luminescence was measured on F-7000 FL spectrophotometer (Hitachi, Tokyo, Japan). UV-visible (UV-vis) spectra were determined via UV2600 spectrometer (Shimadzu, Kyoto, Japan).

### 3.2. UV-Visible Titrations

DNA affinities of synthesized compounds were determined by UV-vis titrations. Generally, a constant volume of CT-DNA solution was added into 3 mL solutions of complexes **1** or **2** (20 μM) in tris buffer (pH = 7.2, 50 mM NaCl, 5 mM Tris) until the absorption intensity in MLCT (metal-ligand charge transfer) band did not decrease. The concentration of CT-DNA in base pairs was measured using the absorbance of DNA solution at 260 nm using molar extinction coefficient (ε = 13,200 M^−1^·cm^−1^) [43]. The binding constants of two complexes (*K*) were calculated by McGhee-Hippel Equation (1a,b) [23].

(1a) $$(\varepsilon_{a}-\varepsilon_{f})/(\varepsilon_{b}-\varepsilon_{f})=(b-{\left(b^{2}-2K^{2}C_{t}[\mathrm{DNA}]/s\right)}^{1/2})/2KC_{t}$$

(1b) $$b=1+KC_{t}+K[\mathrm{DNA}]/2s$$

where *ε_f_*, *ε_a_*, and *ε_b_* are the molar absorptivity for the free complex, DNA-bound complex, and DNA-saturated complex, respectively. *C_t_* is concentration of complex. *s* is the binding site size.

### 3.3. Viscosity Measurement

DNA viscosities were obtained by measuring the flow time of buffer, DNA, and DNA-bound solutions of ruthenium complexes with an Ubbelohde viscometer. Every sample was tested three times at 30.0 ± 0.1 °C. The values of viscosity werecalculated by using the equation *η*_i_ = (*t*_i_ − *t*_0_)/*t*_0_, where *η*_i_ is the viscosity of the sample, and *t*_i_ and *t*_0_ are the flow time of the sample and buffer solution. The change in viscosity was presented by the plot of (*η*/*η*_0_)^1/3^ against binding ratio, where *η* is the viscosity of the sample for DNA-Ru solution, and *η*_0_ is the viscosity for DNA solution without complex [24,25,26].

### 3.4. Emission Measurements

Emission titrations were performed by adding DNA solution into 1 mL complex solution (5 μM) in tris buffer. For emission quenching experiments, [Fe(CN)_6_]^4−^ was titrated into Ru(II) complex solutions with or without DNA. After 5 min-incubation, luminescence data from 500 to 800 nm were obtained with the excitation wavelength at 450 nm. The slit for excitation or emission was 5 nm.

Ethidium bromide (EB) displacement experiments were measured by emission spectra with the excitation wavelength at 515 nm. The solution of Ru(II) complex was added into EB-DNA mixed solution. The data were recorded from 530 to 750 nm.

Singlet oxygen yield of the two complexes was determined by the fluorescence measurements for the mixed solutions of ruthenium complexes (20 μM) and DPBF (20 μM) (1,3-Diphenylisobenzofuran) in methanol under irradiation at 450 nm. The spectra were recorded with excitation at 405 nm.

### 3.5. DNA Photocleavage Experiments

DNA cleavages under irradiation were checked using agarose electrophoresis in TBE buffer. Firstly, pBR322 DNA (30 mM) was mixed with various concentrations of complexes. Then the mixed solutions were irradiated under 365 nm for 1.5 h. After agarose electrophoresis, the gel was stained by EB solution. The photograph was obtained using Tanon 2500R gel imaging systems.

### 3.6. Molecular Docking

Optimized geometry of complex in ground state was obtained using G09 package of program. DFT-B3LYP method and the mixed basis set (6-31G* for C, N, O, H, and SDD for Ru) were used in the theoretical calculation. AutoDock 4.2 package was used to obtain the DNA docking conformation of the complex. The structure of DNA (PDB: 4e7y) was obtained from a protein data bank.

### 3.7. Photocytotoxicity

Cytotoxicity experiments were carried out in in a 96 wells plate. The selected cells included Hela, A549, and A375 cells. Ruthenium complexes or cisplatin were added into the cells and incubated for 12 h in the dark. The culture medium was then replaced with fresh medium. After 10 min irradiation for the cells (LED system 450 nm, 6 mW/cm^2^), PBS was replaced with DMEM/10% FCS, followed by an additional 36 h of incubation. IC_50_ values were determined by using standard MTT method.

### 3.8. Synthesis

#### 3.8.1. 2-(4′-Methyl-bipyridine-4-yl)-1H-imidazo[4,5-b]Naphthalene (Mbin)

4′-Methyl-2,2′-bipyridine-4-carboxaldehyde [44] (0.198 g) and 2, 3-diaminonaphthalene (0.158 g) were dissolved in 15 mL dimethylacetamide. Then, NaHSO_3_ (0.208 g) was added into the solution. After reflux for 4 h, the addition of 200 mL water into the cooled solution gave a dark brown precipitate. After filtration and water wash, the product was obtained. Further purification was performed by recrystallization in ethanol. Yield: 0.158 mg, 39.3%. Anal (%): (Found: N, 16.52; C, 78.67; H, 4.81%. Calcd for C_22_H_16_N_4_: N, 16.66; C, 78.55; H, 4.79%). ESI-MS: *m*/*z* = 336.2 ([M+1]^+^). ^1^H NMR (400 MHz, ppm, DMSO-d_6_): 13.52 (s, 1H), 9.26 (s, 1H), 8.92 (d, 1H, J = 4.0 Hz), 8.66 (d, 1H, J = 4.0 Hz), 8.34 (s, 2H), 8.28 (d, 2H, J = 4.0 Hz), 8.06 (m, 2H), 7.43 (m, 2H), 7.38 (d, 1H, J = 4.0 Hz), and 2.47 (s, 3H).

#### 3.8.2. Synthesis of Ruthenium Complexes

The suspension of [Ru(L)_2_Cl_2_]·2H_2_O (L = phen or bpy) (0.4 mmol) and mbin (0.4 mmol, 0.156 g) in ethylene glycol (10 mL) was refluxed for 4 h under argon. After the reaction was completed, 100 mL water and solid KPF_6_ were added to give the dark red precipitate. After filtration and dryness in vacuo, the product was obtained by neutral aluminum oxide column with 20% toluene in acetonitrile.

##### [Ru(bpy)_2_(mbin)](PF_6_)_2_(**1**)

Yield: 36.4%. (Found: N, 10.69; C, 48.46; H, 3.06%. Calcd for C_4__2_H_32_N_8_F_12_P_2_Ru: N, 10.78; C, 48.52; H, 3.10%). ESI-MS (CH_3_CN): *m*/*z* = 374.6 ([M-2PF_6_^−^]^2+^). ^1^H NMR (400 MHz, ppm, DMSO-d_6_): 13.47 (s, 1H), 9.49 (s, 1H), 8.86 (t, 5H, J_1_ = 9.2 Hz, J_2_ = 7.6 Hz), 8.36 (d, 1H, J = 4.0 Hz), 8.20 (m, 6H), 8.07 (d, 2H, J = 8.0 Hz), 7.98 (d, 1H, J = 5.6 Hz), 7.90 (d, 1H, J = 5.6 Hz), 7.76 (m, 3H), 7.62 (d, 1H, J = 6.0 Hz),7.57 (m, 4H), 7.35 (d, 3H, J = 6.4 Hz), and 2.62 (s, 3H). ^13^C NMR (101 MHz, ppm, DMSO-d_6_): 158.02, 157.06, 156.92, 156.13, 152.66, 151.92, 151.76, 151.56, 150.94, 150.50, 144.30, 138.46, 137.98, 135.89, 131.57, 130.60, 129.50, 128.78, 128.41, 128.01, 125.92, 124.98, 124.61, 124.10, 121.44, 117.00, 107.95, and 21.28.

##### [Ru(phen)_2_(mbin)](PF_6_)_2_ (**2**)

Yield: 33.6%. Anal (%): (Found: N, 10.24; C, 50.64; H, 3.02%, Calcd for C_46_H_32_N_8_F_12_P_2_Ru: N, 10.30; C, 50.79; H, 2.97%). ESI-MS (CH_3_CN): *m*/*z* = 398.8 ([M-2PF_6_^−^]^2+^). ^1^H NMR (400 MHz, ppm, DMSO-d_6_): 13.46 (s, 1H), 9.52 (s, 1H), 8.85 (t, 5H, J_1_ = 9.2 Hz, J_2_ = 7.6 Hz), 8.41 (m, 6H), 8.31 (d, 1H, J = 1.2 Hz), 8.17 (s, 1H), 8.07 (m, 3H), 7.96 (m, 5H), 7.74 (dd, 2H, J_1_ = 1.2 Hz, J_2_ = 1.2 Hz), 7.59 (d, 1H, J = 0.8 Hz), 7.45 (t, 2H, J1 = 0.8 Hz, J2 = 0.8 Hz), 7.34 (d, 1H, J = 0.4 Hz), and 2.61 (s, 3H).^13^C NMR (101 MHz, ppm, DMSO-d_6_): 158.39, 156.48, 153.17, 153.02, 152.81, 151.94, 151.45, 150.44, 147.68, 147.57, 147.43, 147.23, 144.28, 137.94, 137.34, 135.88, 131.56, 131.01, 130.95, 130.89, 130.59, 129.36, 128.78, 128.56, 128.01, 126.89, 125.80, 125.09, 124.44, 124.10, 121.33, 116.98, 107.94, and 21.26.

## 4. Conclusions

Two naphthyl-appended ruthenium complexes were synthesized and evaluated for DNA binding properties, DNA photocleavage abilities, and photocytotoxicity. DNA binding investigations revealed that two complexes displayed intercalation with calf thymus DNA. They can produce singlet oxygen under 365 nm light, which led to photo-reduced DNA cleavage. Furthermore, two complexes displayed obvious photocytotoxicity and large PI values against selected cell lines, which indicated that two complexes presented the potential PDT activities. In addition, complex **2** displayed larger PI values against all cell lines than complex **1**, which was attributedto its higher singlet oxygen quantum yields and stronger DNA binding affinity.

## Figures and Tables

**Table 1:** The IC_50_ and PI values for two complexes against tumor cells.

Complex	IC_50_ (μM)								
	HeLa			A549			A375		
	Dark	Light	PI	Dark	Light	PI	Dark	Light	PI
**1**	>100	0.95 ± 0.21	105	>100	0.85 ± 0.26	117	>100	0.48 ± 0.16	208
**2**	>100	0.67 ± 0.15	149	92.67 ± 3.28	0.58 ± 0.06	160	90.34 ± 4.87	0.12 ± 0.01	753
**[Ru(bpy)_3_]^2+^** [20]	>300	161 ± 5.62	1.86	>300	152 ± 4.34	1.97			
**[Ru(bpy)_2_dppn]^2+^** [42]	110 ± 28	0.39 ± 0.06	282						
**Cisplatin**	45.75 ± 3.31	43.61 ± 5.52	1.05	38.27 ± 2.46	33.81 ± 5.17	1.13	33.86 ± 3.82	30.63 ± 5.47	1.11

## Data Availability

Not applicable.

## Supplementary Materials

The following are available online at https://www.mdpi.com/article/10.3390/molecules27123676/s1; Figures S1 and S2. ^1^H NMR and ^13^C NMR of the ligand and its ruthenium complexes in (CD_3_)_2_SO; Figure S3. The optimized geometry of complex **2**; Figure S4. The ds-DNA (PDB: 4E7Y) binding conformation of complex 1 by docking.

## Sample Availability

Samples of the compounds **1** and **2** are available from the authors.
