# Supplementary material for: DNA Interaction, DNA Photocleavage, Photocytotoxicity In Vitro, and Molecular Docking of Naphthyl-Appended Ruthenium Complexes
Source: Molecules. 2022 Jun 8;27(12):3676. doi: 10.3390/molecules27123676 (PMC9227816; doi:10.3390/molecules27123676)
Supplement: Supplementary file 1 [file molecules-27-03676-s001.zip › molecules-1698197-supplementary.pdf]

Supplementary material

DNA interaction, DNA photocleavage, photocytotoxicity in vitro, and molecular docking of naphthyl-appended ruthenium complexes

Xia Hu, Qian Luo, Yao Qin, Yao Wu, and Xue-Wen Liu*

Hunan Provincial Key Laboratory of Water Treatment Functional Materials, Hunan Province Engineering Research Center of Electroplating Wastewater Reuse Technology, College of Chemistry and Materials Engineering, Hunan University of Arts and Science, Changde 415000, China

.

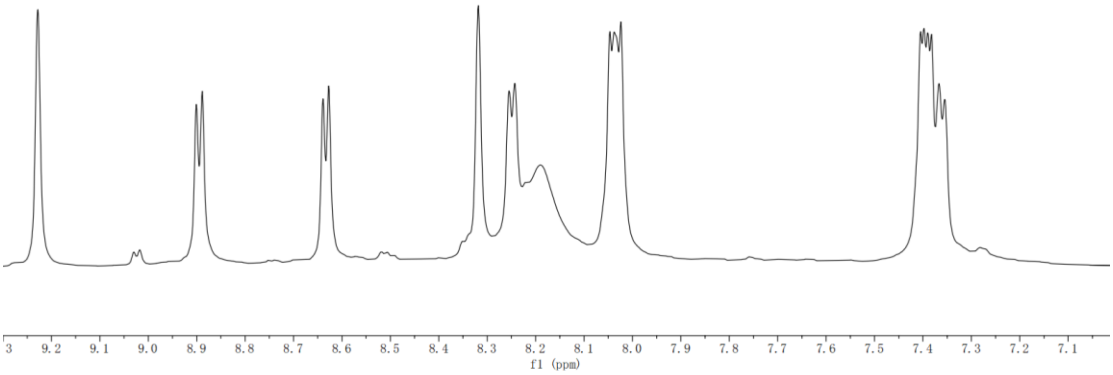

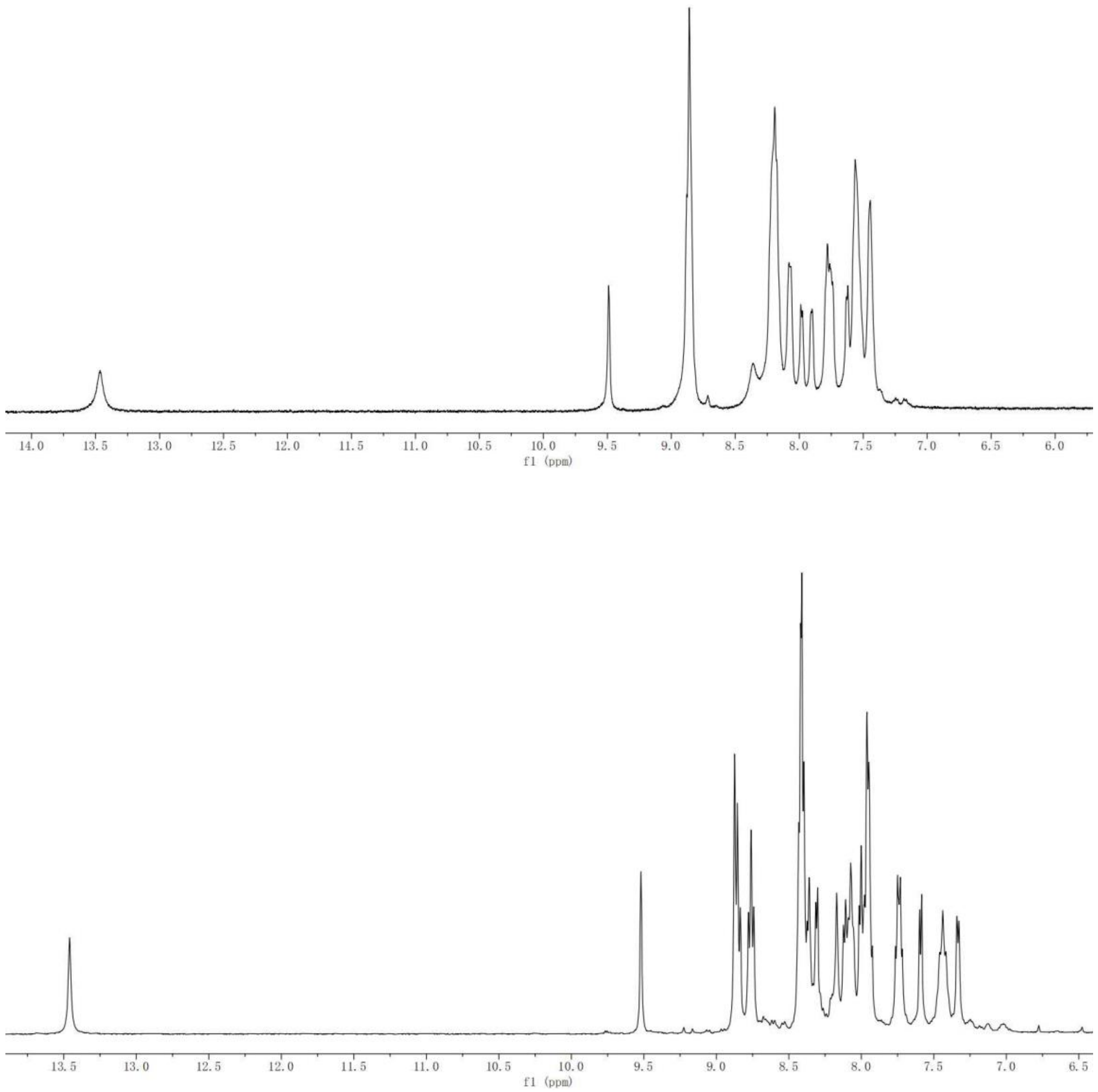

Figure S1. ^{1}H NMR in aromatic region of the ligand mbin (top), complexes **1** (middle), and **2** (bottom) in $(CD_3)_2SO$ (400 MHz)

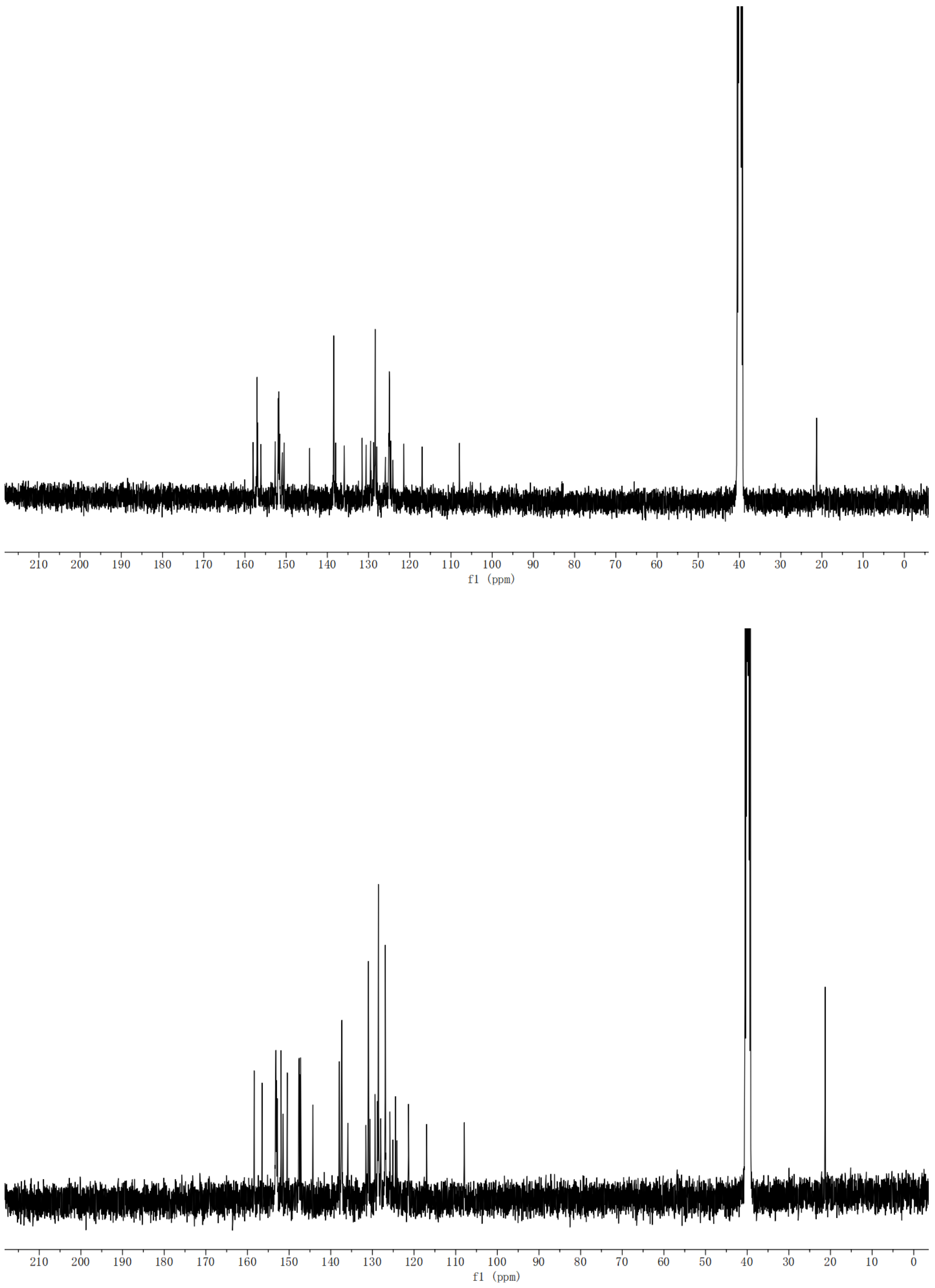

Figure S2. ^{13}C NMR of tcomplexes **1** (top), and **2** (bottom) in $(CD_3)_2SO$ (101 MHz).

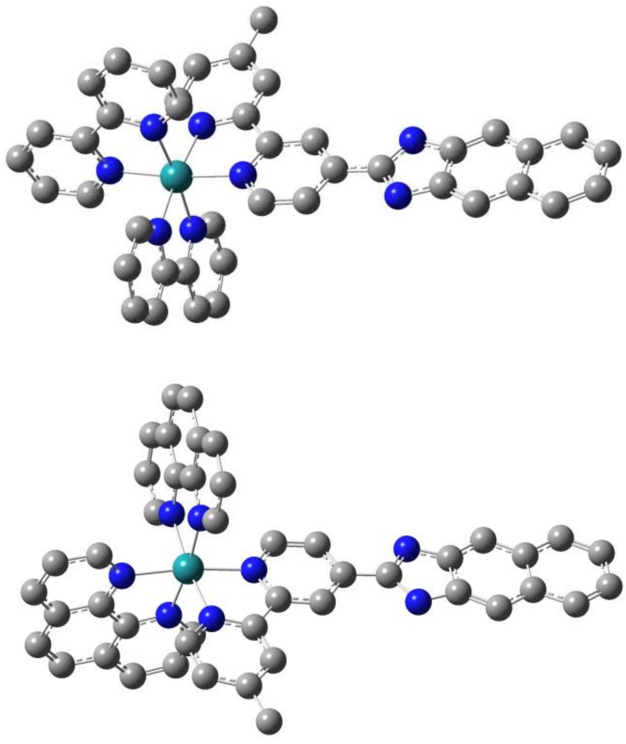

Figure S3. The optimized geometry of complex **1** (top) and **2** (bottom).

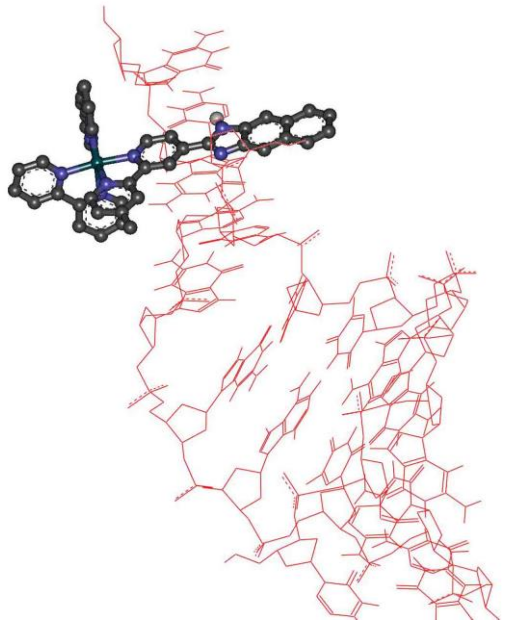

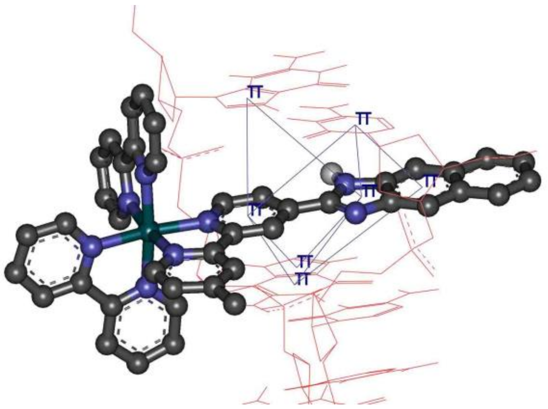

Figure S4. The ds-DNA (PDB: 4E7Y) binding conformation of complex **1** by docking.
